# Construct Validity of the eHealth Literacy Scale (eHEALS) Among Two Adult Populations: A Rasch Analysis

**DOI:** 10.2196/publichealth.4967

**Published:** 2016-05-20

**Authors:** Jennifer Nguyen, Michael Moorhouse, Barbara Curbow, Juliette Christie, Kim Walsh-Childers, Sabrina Islam

**Affiliations:** ^1^ Minority Cancer Research and Training (MiCaRT) Center University of Florida Health Cancer Center Orlando, FL United States; ^2^ University of Florida Department of Behavioral Science and Community Health Gainesville, FL United States; ^3^ University of Maryland Department of Behavioral and Community Health College Park, MD United States; ^4^ University of Florida Department of Journalism Gainesville, FL United States

**Keywords:** eHealth, eHEALS, internet, measurement, rasch, public health

## Abstract

**Background:**

The Internet has become a ubiquitous venue for information seeking, especially for health information. Public health practitioners have noticed the promise and potential of the Internet, however, little is known about individuals' skills of their eHealth literacy. The eHealth Literacy Scale, eHEALS, was designed to measure perceptions of individuals' eHealth literacy skills.

**Objective:**

The objective of the study was to examine the psychometric validity and reliability of the eHEALS with two adult populations using the Rasch Model.

**Methods:**

A college-aged sample and an Internet-based sample (Amazon's MTurk) were recruited to complete the eHEALS, demographic questions, and a health literacy scale. Using WINSTEPS and SPSS, unidimensionality, item fit, rating scale, item hierarchy, person ability-item match, and reliability were analyzed, compared, and contrasted against each sample and to other samples found in the literature.

**Results:**

An exploratory factor analysis supported unidimensionality in both samples. More than 90% of respondents from both samples fit the model. No items were outright misfitting. Both samples separated into three distinct groups.

**Conclusions:**

Based on the results, the eHEALS is a reliable and consistent measurement tool for a college sample and an Internet-based sample. As these individuals are most likely to use the Internet as a health resource, it is necessary to learn and know their skills versus perceiving that they can critically and successfully navigate the Internet. Further analyses are necessary to ensure that the eHEALS can serve as a standard eHealth literacy measure for public health.

## Background

Using the Internet is now a standard practice for people seeking information about health care and health conditions. The PewResearch Internet Project estimates that more than 85% of adults in the United States use the Internet, with nearly three-quarters using the Internet for health information research [1]. Consequently, public health researchers are studying critical issues such as the quality of the Web-based health content and individuals’ ability to navigate the Web and find information [2-6].

Norman and Skinner [7] coined the term “eHealth literacy” to describe the ability to navigate the Internet for health information. Unlike general health literacy, eHealth literacy also considers individual computer and Web navigation skills. Thus, eHealth literacy encompasses a constellation of literacies, including computer literacy, scientific literacy, health literacy, traditional literacy, media literacy, and information literacy. Using this model, Norman and Skinner [8] created the eHealth Literacy Scale (eHEALS) to measure individuals’ perceptions of their own digital health literacy skills [9].

Accurately measuring eHealth literacy is imperative to addressing public health disparities. Many studies have used the eHEALS to measure eHealth literacy despite a lack of psychometric evidence [3-7,10]. When first created, the instrument was tested on a sample of middle school children [9]. Since then, Dutch and Japanese researchers have explored the psychometric properties of the eHEALS; however, both Dutch and Japanese researchers translated the instrument into their own native languages [4,11]. There are no known follow-up attempts to analyze the eHEALS using an English-speaking adult sample.

A 1-parameter logistic item response theory model, the Rasch model, is a mathematical framework created to empirically analyze categorical data [12]. The Rasch model is commonly used within the health professions, social sciences, education field, and market research [9,13-16]. The Rasch perspective examines each item contained in the measure versus examining the items as a conglomerate. Essentially, the Rasch model accounts for the “difficulty” of the item and expects that if a person of average ability were to accomplish a task of average difficulty, the person should have a high probability of accomplishing the “easier” tasks as well. The simplest Rasch formula is: log [*P*_n_/1‑ *P*_n-1_]=B_n_−D_1_, where *P*_n_=probability of person n responding to item *i* correctly, *P*_n_=probability of person n responding to item *i* incorrectly, B_n_=trait/ability level of person n, and D_1_=difficulty of item *i* [17].

In this study, the construct validity of the eHEALS was analyzed among 2 adult samples—university students and adults who use the Internet. The following constructs were investigated: (1) unidimensionality, (2) fit of items and participants, (3) item rating structure, (4) item difficulty hierarchy, and (5) person ability-item difficulty match.

## Methods

### Instrument: eHEALS

Created to measure a combination of comfort, knowledge, searching, evaluation, and application skills, eHEALS was developed as a self-reporting tool that can be administered by any health professional with little to no training [9]. Items reflect conceptualizations of the 6 key eHealth literacy constructs, and specialists were contacted for their expert feedback, whereas youth in TeenNet Research provided their views on readability and relevance [9]. After pilot testing with 89 teenagers and young adults, the instrument was finalized into its 8-item form (see Multimedia Appendix 1).

Validated with a middle school sample (n=664, mean age 14.95 years) in Canada, the analysis revealed an *α* =0.88 with item-scale correlations ranging from *r* =0.51 to 0.76. A principal component analysis found a single-factor solution, with factor loadings from 0.60 to 0.84 among the 8 items [9]. All questions use a 5-point Likert scale ranging from strongly disagree to strongly agree. An exploratory factor analysis conducted on a modified 6-item version of the eHEALS on an adult, Israeli sample (n=1289) produced similar factor loadings (.62 to .84) among the items. The item-scale correlation ranges from r=0.51 to 0.76. The coefficient alpha was lower (*α* =0.86) but similar to that in reported results [9]. The principal components analysis also revealed a single-factor solution [6]. Neter et al. conducted a confirmatory factor analysis on their modified eHEALS, alongside a few other measures that they used in their study, including outcomes perception, Internet access, and digital literacy [6]. They found that the scales were independent of each other via a 2-model fit analysis. Other psychometric evaluations have been conducted; however, they have been on translated versions of the eHEALS [11,16,18,19].

### Recruitment and Participants

The first adult sample was obtained through a convenience sampling of college students. Undergraduate students enrolled in a health science research methods course in a large, southern university completed a questionnaire comprising the eHEALS in addition to questions pertaining to knowledge, attitudes, and beliefs. The questionnaire was used to demonstrate the process of informed consent, the various types of questions in psychosocial research, and how researchers analyze data. Inclusion criteria for eligible participants consisted of being 18 years of age or older, registered for the course, being present on the day of data collection, and agreeing to participate in the data collection. Results from this sample are in Table 1.

The second adult sample was acquired through Amazon’s Mechanical Turk (MTurk), a crowdsourced Internet marketplace, wherein individuals and/or businesses can ask people to perform tasks that computers cannot complete. Requesters post various tasks, known as human intelligence tasks (HITs), for individuals to choose and complete. Some HITs often involve transcription requests, translation requests, market survey research, opinion essays, and social science research. Individuals who complete these tasks are known as workers or providers or turkers and are compensated for their time [20].

Despite being a relatively new presence within social science research, MTurk appears to deliver reliable and usable user data. Several studies demonstrate that there are almost no differences in effect sizes when compared to other convenience samples. In addtion, samples from turkers are as reliable as other samples collected from the Internet. There are no statistical differences between in-laboratory or field samples, and samples from turkers tend to be more diverse than other Internet samples [21-23].

To access the HIT for this study, turkers get qualified if their HIT approval rate percentage was ≥98 with at least 500 completed and approved HITs. These scores are based on past performance ratings given by requesters. Those turkers who fail to follow instructions have their approval rating lowered. This stipulation was desgined to ensure that only individuals with MTurk familiarity and a good work history could participate in the data collection. Turkers had to first accept the task and then consent to being a part of the study. Results from this sample are presented under Study 2.

### Data Analysis

#### Unidimensionality

A critical assumption in item response theory models, including Rasch, predicates on unidimensionality, which refers to the focus of the measure and its ability to focus on one variable at a time [12]. An exploratory components analysis using SPSS [24] was conducted. Eigenvalues and a visual inspection of the scree plot determined the number of extracted factors.

#### Item Fit

Using infit and outfit statistics, the fit to the model was analyzed. infit statistics are sensitive to data that are related to the items, whereas outfit statistics represent the relationships between data that are not related to the item (or person). The ideal fit statistic is 1.0, as fit is determined by calculating observed variance over expected variance [15]. Because the eHEALS is a survey of lower stakes (ie, the results of the survey do not have direct or definite consequences for the test-taker), the acceptable range of fit statistics is 0.6-1.4 [25]. An infit value of 0.6 indicates that 40% less variation was observed than modeled and a value of 1.4 indicates that 40% more variation was observed than modeled [12]. Mean-squares below the threshold overfit the model and thus suggest the data are more predictable than expected. Conversely, mean-squares above the threshold underfit the model, suggesting that the data are less predictable than expected. The second criterion of fit is the standardized *t* score, represented as the ZSTD by Winsteps. ZSTD scores examine the probability of significance that the data fit the Rasch model, determining the actual fit versus the theorized fit based on the model (observed vs expected). The acceptable range for ZSTD scores is ±2.0 [15]. Consequently, for an item or a person to misfit, the mean-square must be outside of the range of 0.6-1.4 as well as exceed the acceptable range for ZSTD.

#### Rating Scale

Although Linacre outlines 10 guidelines for rating scale optimization, he stresses the following 3 as essential critieria[17]: first, each rating category must have at least 10 observations. Linacre determined that without 10 observations for each rating category, a stable estimation of threshold value cannot be calculated, suggesting that the category may be unnecessary to measure . Second, average calibrations advance monotonically, meaning that on average, individuals with stronger ability should respond to higher categories, whereas individuals with lower ability should respond to lower categories. Lack of monotonicity strengthens the call for collasping categories. The third essential criterion stipulates that the outfit mean-squares be less than 2.0 for each rating category. Values greater than 2.0 indicate that there is unnecessary noise and misinformation in that particular category [17].

#### Item Hierarchy, Person Ability-Item Match, and Reliability

The Rasch model allows inferences to be made about a individual ability with regard to the difficulty of the items. For instance, a person with a high math ability level should have a higher probability of answering more difficult questions correctly than a person with lower math ability. Similarly, more difficult items are less likely to be answered correctly than easier items [19]. The analysis revealed the order of item difficulty, ranked from easiest to hardest items. In addition, Rasch analysis allows the researcher to examine how well the ability of the sample matches the difficulty of the items. Person reliability (similar to Cronbach’s alpha) estimates how well a measure can separate individuals on the construct. Conversely, person separation determines the strata or distinct levels that individuals are “spread” out on the measured construct.

## Results

### Study 1

#### Sample

In total, 164 students took the survey. Of the respondents, 20% (n=33) were male, and 80% (n=131) were aged between 18-34 years, with 83.6% of the students being aged 20 or 21 years. Almost 72% (n=118) of students reported that they spent more than 3 hours each day on the Internet, 25.6% (n=42) of students reported only 1-3 hours on the Internet, and less than 3% (n=4) reported spending less than an hour daily online [18]. Table 1 displays the demographic summary.

#### Unidimensionality

An ECA revealed that only one factor had an eigenvalue greater than 1. The scree plot showed one “bend,” and the factor score matrix only extracted one factor, which supported the assumption of unidimensionality.

#### Rating Scale Analysis

The most common criteria violation was failing to have at least 10 observations for each rating category. Few respondents chose “strongly disagree” and “disagree.” There were 2 instances in which the outfit mean-squares were outside the range of +2.0; the outlier could be due to the low observations in those rating categories. Table 2 presents the categories for each item that violated the essential criteria.

#### Model Fit

Fit order is presented in Table 3. All the items met the criteria for both infit and outfit. Ninety-five percent of participants (155 of 163) fit the model. Eight (n=8) participants violated both infit and outfit criteria.

#### Precision

The Rasch model’s equivalency of Cronbach’s alpha is person reliability, which was 0.80. Person separation was 2.02, indicating that the eHEALS separated the sample into 3.03 strata or 3 distinct groups.

#### Person Ability Item-Difficulty Match

**Figure 1:**
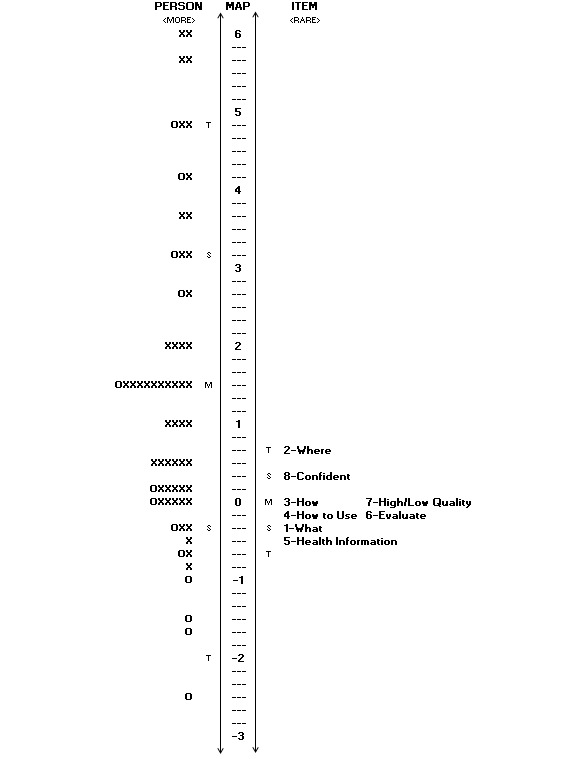
Person ability item-difficulty match of the college sample. Persons are on the left of the line, whereas the item difficulty map is to the right of the line. Each “O” represents 1-2 individuals, whereas each “X” represents 3 persons.

Figure 1 is the map of item difficulty contrasted with person ability. Person ability (on the left side of the line) is presented from the highest ability (top) to the lowest ability (bottom). Items, on the right side of the line, are ranked from easiest (bottom) to hardest (top). Although there were no floor effects, there was a ceiling effect, with 6 individuals. Thus, the eHEALS was incapable of measuring individuals of extremely high ability.

### Study 2

#### Sample

A total of 366 individuals took the survey. More than half of the participants were males (n=203), leaving a total of 159 female respondents. Almost 59% (n=210) of the individuals were aged 18-32 years. The age range of participants captured a wider group; some participants indicated being agedolder than 65 years.Eleven percent (n=40) of participants reported being online only 1-3 hours a day; 33% (n=120) of participants reported spending 4-6 hours online daily; and approximately 26% (n=94) of respondents spend a reported 7-10 hours online daily. Table 4 displays the demographic summary.

#### Unidimensionality

Similar to the results from Study 1, an EFA showed that only one factor was extracted, suggesting one latent variable or factor.

#### Rating Scale Analysis

Paralelling Study 1 outcomes, the most common essential guideline violation was not having 10 observations in each rating category. In addition, items 3 and 5 violated all essential criteria. Table 5 shows where all violations occurred.

**Table 1:** Demographics of respondents: study 1 (n=164).

Demographic		N	%
Sex		
	Male	33	20.1
	Female	131	79.9
Age		
	18-24	158	96.3
	25-32	5	3.0
	33-39	1	0.7
Time online/day		
	<1 hour	4	2.4
	1-3 hours	42	25.6
	>3 hours	118	72.0

**Table 2:** Ratings that violate essential criterion: study 1.

Item	Observed count^a^	Monotonicity^b^	Outfit^d^
I know what health resources are available on the Internet.	1-(SD)^d^, 8-(D)		
I know where to find helpful health resoures on the Internet.	0-(SD)		
I know how to find helpful health resources on the Internet.	0-(SD), 6-(D)		
I know how to use the Internet to answer my questions about health.	0-(SD), 5-(D)		
I know how to use the health information I find on the Internet to help me.	1-(SD), 7-(D)		4.12-(SD)
I have the skills I need to evaluate the health resources I find on the Internet.	2-(SD), 7-(D)		
I can tell high-quality health resources from low-quality health resources on the Internet.	3-(SD)		
I feel confident in using information from the Internet to make health decisions.	1-(SD)		

^a^The numbers in the “Observed count” column are the counts of each answer choice in violation of the essential criterion.

^b^Because none of the items violated montoncity, no data are reported in that column.

^c^The numbers in the “Outfit” column are the values of the misfitting outfit means-square.

^d^SD: strongly disagree, D: disagree.

**Table 3:** Item fit: study 1 (college sample)^a^.

			Infit		Outfit	
Item	Measure	Model SE	MNSQ	ZSTD	MNSQ	ZSTD
I know what health resources are available on the Internet.	−0.34	0.14	0.89	−0.80	0.8	−1.4
I know where to find helpful health resoures on the Internet.	0.74	0.15	0.84	−1.20	0.79	−1.2
I know how to find helpful health resources on the Internet.	0.06	0.16	1.16	1.20	1.12	0.9
I know how to use the Internet to answer my questions about health.	−0.16	0.15	0.99	0.0	0.91	−0.5
I know how to use the health information I find on the Internet to help me.	−0.54	0.14	0.90	−0.60	0.88	−0.7
I have the skills I need to evaluate the health resources I find on the Internet.	−0.15	0.14	1.06	0.50	1.05	0.4
I can tell high-quality health resources from low-quality health resources on the Internet.	0.07	0.12	1.16	1.20	1.12	0.9
I feel confident in using information from the Internet to make health decisions.	0.31	0.13	1.15	1.40	1.2	1.8

^a^This is a table showing item statistics and the fit of each of the items. There were no infit or outfit violations. The infit statistics are weighted to the performance of persons close to the item value. These individuals give a sensitive insight into the item’s performance. The outfit statistics are not weighted and are not sensitive to the influence of outlying scores.

**Table 4:** Demographics of respondents: study 2 (n=366).

Demographics		N	%
Sex			
	Male	203	55.5
	Female	159	43.4
Age			
	18-24	47	12.9
	25-32	163	45.0
	33-39	71	19.5
	40-46	31	8.6
	46-52	23	5.2
	53-59	17	4.7
	60-64	10	2.8
	>65	4	1.1
Time online/day			
	1-3 hours	40	11.0
	4-6 hours	120	33.1
	7-10 hours	94	25.9
	11-13 hours	36	9.9
	14-16 hours	20	5.5
	>17 hours	6	1.7
Education			
	8th grade	1	0.3
	Some high school, no diploma	4	1.1
	HS diploma or equivalent	44	12.2
	Some college, no degree	75	20.7
	Trade/technical/vocational training	13	3.6
	Associate’s degree	42	11.6
	Bachelor’s degree	148	40.9
	Master’s degree	28	7.7
	Professional degree	5	1.4
	Doctorate degree	2	0.6

**Table 5:** Ratings that violate essential criterion: study 2.

Item	Observed count^a^	Monotonicity^b^	Outfit^c^
I know what health resources are available on the Internet.^d^	2-(SD)^e^	(D)^f^	(SD)-9.90
I know where to find helpful health resoures on the Internet.	1-(SD)		
I know how to find helpful health resources on the Internet.	0-(SD)		
I know how to use the Internet to answer my questions about health.	0-(SD), 2-(D)		
I know how to use the health information I find on the Internet to help me.^d^	1-(SD), 6-(D)	(D)	(SD)-6.83
I have the skills I need to evaluate the health resources I find on the Internet.	1-(SD)		
I can tell high-quality health resources from low-quality health resources on the Internet.			
I feel confident in using information from the Internet to make health decisions.	3-(SD)		

^a^The numbers in the “Observed count” column are the counts of each answer choice in violation of the essetia criterion.

^b^There were 2 instances where “disagree” did not advance motonically.

^c^The numbers in the “Outfit” column are the values of the misfitting Outfit means-squares, including where the violation occur.

^d^This indicates violations of all essential criteria.

^e^SD: strongly disagree.

^f^D: disagree.

**Table 6:** Item fit: study 2

Item	Measure	Model SE	Infit^b^		Outfit^c^	
MNSQ	ZSTD	MNSQ	ZSTD
I know what health resources are available on the Internet.	−0.18	0.11	1.27	2.5^a^	1.42^a^	3.0^a^
I know where to find helpful health resoures on the Internet.	−0.56	0.11	0.91	−0.9	0.71	−2.4^a^
I know how to find helpful health resources on the Internet.	0.51	0.12	0.83	−1.8	0.70	−2.5^a^
I know how to use the Internet to answer my questions about health.	0.16	0.12	0.78	−2.5^a^	0.64	−3.5^a^
I know how to use the health information I find on the Internet to help me.	−0.58	0.12	0.79	−2.1^a^	0.77	−2.0
I have the skills I need to evaluate the health resources I find on the Internet.	−0.57	0.11	0.98	−0.1	0.9	−1.0
I can tell high-quality health resources from low-quality health resources on the Internet.	0.99	0.10	1.21	2.5^a^	1.25	2.6^a^
I feel confident in using information from the Internet to make health decisions.	0.25	0.10	1.04	0.5	1.00	0.00

^a^It denotes violation of model fit; no items violated all criteria.

^b^Infit statistics are weighted to the performance of persons close to the item value. These individuals give a sensitive insight into the item’s performance.

^c^Outfit statistics are not weighted and are not sensitive to the influence of outlying scores.

**Figure 2:**
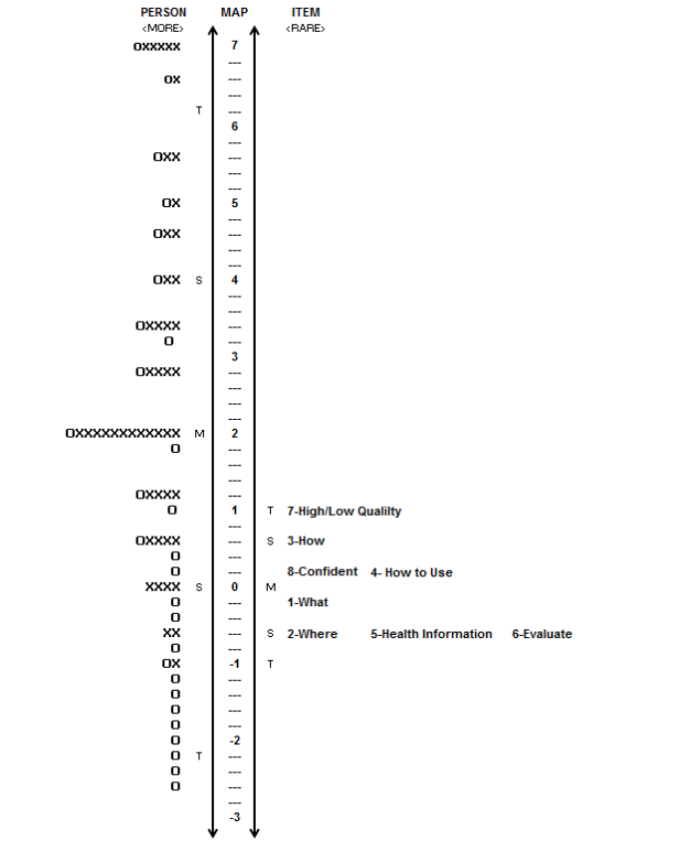
Person ability of the MTurk sample is on the left side, whereas item difficulty is on the right side. Each “O” represents 1-2 individuals, whereas each “X” represents 3 persons.

#### Model Fit

Table 6 displays the corresponding values for model fit. Although there are violations of outfit criteria (eg, item 1), no items violated both infit and outfit. Almost 93% of respondents fit the model, with 27 individuals violating both infit and outfit criteria.

#### Precision

The person reliability was 0.81, whereas person separation was 2.07. The eHEALS separated the sample into 3.07 separate strata.

#### Person Ability Item-Difficulty Match

The map of item difficulty and person ability is presented in Figure 2. Like Study 1, person ability is on the left side of line, with individuals with higher levels of ability on top. Item difficulty is on the right side of the line, with more difficult items on top. Approximately 8.3%-9.7% of the sample had ability levels that eHEALS could not capture (n=30-35).

## Discussion

### Principal Findings

Overall, eHEALS is a reliable and consistent measurement tool for perceived measurement of eHealth literacy. An exploratory factor analysis showed that items loaded on a single factor solution, thereby supporting the criterion of unidimensionality. More than 90% of respondents from both samples fit the model. Although some items violated either infit or outfit guidelines, there were no outright misfitting items. Furthermore, the discordance between the mean of person ability and the mean of item difficulty was assumed as we sampled from a college population and a younger generation. The analysis separated both samples into 3 distinct groups, but further analyses are needed to describe the groups.

As eHEALS measures individuals’ level of eHealth literacy, a small ceiling effect and no floor effect both occurred, as expected. The eHEALS did not adequately measure every participant’s ability level. The item map only showed a spread of 2 logits, whereas person ability level spread over multiple logits. Furthermore, there are limitations in the eHEALS’ rating scale, as evident in the ratings that violated the essential criteria as outlined in Linacre [18]. The violations were due to the low number of observations (less than 10) in the lower parts of the rating scale (ie, the strongly disagree and disagree choices).

**Figure 3:**
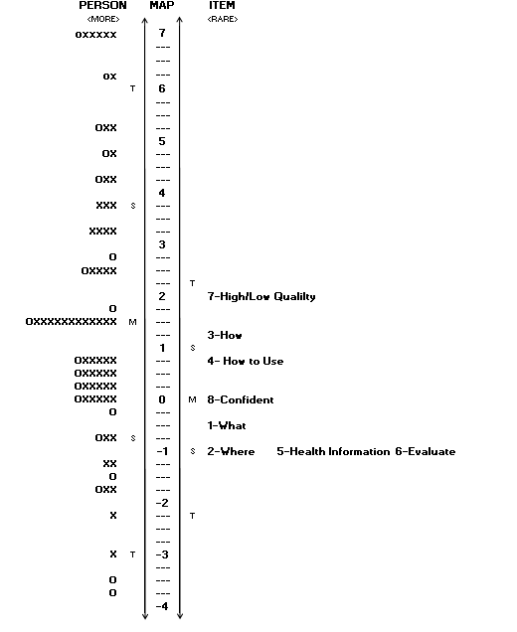
The person and item map after the rating scale was collapsed. Person ability is on the left side, whereas item difficulty is on the right side. Each “O” represents 1-2 individuals and each “X” is equal to 3 persons.

It was hypothesized that it may be beneficial to collapse “strongly disagree” and “disagree” together, to avoid violating essential guidelines. For the MTurk sample, collapsing the 2 categories did not change person reliability (0.81) and remained to separate the sample into 3.09 distinct strata. As demonstrated in Figure 3 , a ceiling effect is still present; however, item difficulty is more spread out, approximately over an additional half logit. Moreover, the means between person ability and item difficulty are approximately one and a half logits away from each other. In contrast, before combining “strongly disagree” and “disagree,” the two means were approximately two logits away from each other. Although further analysis should be conducted to ensure that there is no loss of validity and reliability, the reduction of the rating scale may relieve some test-taking burden and separate persons and items more distinctly.

Although the item difficulty map was similar between the 2 samples, some subtle differences exist. For instance, the college sample rated “I know where to find helpful health resources on the Internet” to be the easiest item and “I know how to use the health information I find on the Internet to help me” to be the hardest item. For the turkers, the easiest item was “I can tell high quality health resources from low quality health resources on the Internet,” whereas the hardest item was “I have the skills I need to evaluate the health resources I find on the Internet.” These differences could be attributed to the demographic make-up of each sample group. The college students are health science students and may therefore be more familiar with the location of health resources on the Internet. With higher education level in the turkers' sample, it may be plausible that they possess higher perceptions of their own ability to distinguish high-quality health information versus low-quality health information.

Knowledge of person ability and item difficulty is strongly relevant, as many public health organizations and doctors communicate with clients and patients online. With constant and easy access to the Internet, health care entities can use the information to tailor their materials and provide effective public health interventions to their targeted audience. For instance, community health workers can use outreach measures to those individuals with lower eHealth literacy by illustrating the differences between a verified Web resource and a blog with questionable health advice, thereby refining individuals’ skills in identifying reliable and accurate online sites.

### Limitations

This analysis bears some limitations. The college sample answered the eHEALS via paper and pencil method. Although Norman and Skinner also administered the eHEALS using paper and pencil, it may be more appropriate to have individuals take the instrument using a mobile or an Internet-connected device [9]. In addition, the college sample covered a somewhat homogeneous group. These students were in a core research methods class that required the usage of the Internet to find health information. Accordingly, their online searching abilities were crucial to their success in the course. Moreover, although involving turkers is novel, the sample cautions the generalizability of the study. Millennials are becoming the largest living generation, yet the higher rates in numbers are attributed to immigrants [26]. Turkers are a special subset of individuals; knowledge of the site, signing up on the site, and completion of a number of tasks were necessary conditions for verification of survey participation.

It is important to note that the combination of the 2 samples represents a large number of millenials in the United States. As young adults and minorities are liklier than any other group to have mobile Internet access, the Internet can serve as a valuable public health tool to improve the health of young adults and minorities in this country [1]. Using the Internet to improve behavioral change outcomes has been shown to be fruitful, especially among such vulnerable populations [27,28]. The productive potential of using the Internet is evident. Now, it is a public health imperative to study eHealth literacy measurement to maximize both the potential impact and reach that the Internet can have on our populaces.

## Appendix

Multimedia Appendix 1 eHEALS: eHealth Literacy Scale.
