# Supplementary material for: Construct Validity of the eHealth Literacy Scale (eHEALS) Among Two Adult Populations: A Rasch Analysis
Source: JMIR Public Health Surveill. 2016 May 20;2(1):e24. doi: 10.2196/publichealth.4967 (PMC4909391; doi:10.2196/publichealth.4967)
Supplement: Multimedia Appendix 1 [file publichealth_v2i1e24_app1.pdf]

eHEALS: eHEALTH LITERACY SCALE

1. I know **what** health resources are available on the Internet.
Strongly Disagree
Disagree
Undecided
Agree
Strongly Agree

2. I know **where** to find helpful health resources on the Internet.
Strongly Disagree
Disagree
Undecided
Agree
Strongly Agree

3. I know **how** to find helpful health resources on the Internet.
Strongly Disagree
Disagree
Undecided
Agree
Strongly Agree

4. I know **how to use** the Internet to answer my questions about health.
Strongly Disagree
Disagree
Undecided
Agree
Strongly Agree

5. I know how to use **the health information** I find on the Internet to help me.
Strongly Disagree
Disagree
Undecided
Agree
Strongly Agree

6. I have the skills I need to **evaluate** the health resources I find on the Internet.
Strongly Disagree
Disagree
Undecided
Agree
Strongly Agree

7. I can tell **high quality** health resources from **low quality** health resources on the Internet.
Strongly Disagree
Disagree
Undecided
Agree
Strongly Agree

8. I feel **confident** in using information from the Internet to make health decisions.
Strongly Disagree
Disagree
Undecided
Agree
Strongly Agree
